# Subcortical Brain Volumes and Neurocognitive Function in Children With Perinatal HIV Exposure: A Population-Based Cohort Study in South Africa

**DOI:** 10.1093/ofid/ofae317

**Published:** 2024-07-17

**Authors:** Catherine J Wedderburn, Shunmay Yeung, Nynke A Groenewold, Andrea M Rehman, Sivenesi Subramoney, Jean-Paul Fouche, Shantanu H Joshi, Katherine L Narr, Nadia Hoffman, Annerine Roos, Diana M Gibb, Heather J Zar, Dan J Stein, Kirsten A Donald

**Affiliations:** Department of Paediatrics and Child Health, Red Cross War Memorial Children's Hospital, University of Cape Town, Cape Town, South Africa; Department of Clinical Research, London School of Hygiene & Tropical Medicine, London, United Kingdom; The Neuroscience Institute, University of Cape Town, Cape Town, South Africa; Department of Clinical Research, London School of Hygiene & Tropical Medicine, London, United Kingdom; The Neuroscience Institute, University of Cape Town, Cape Town, South Africa; Department of Psychiatry and Mental Health, University of Cape Town, Cape Town, South Africa; Medical Research Council Tropical Epidemiology Group, London School of Hygiene & Tropical Medicine, London, United Kingdom; Department of Paediatrics and Child Health, Red Cross War Memorial Children's Hospital, University of Cape Town, Cape Town, South Africa; The Neuroscience Institute, University of Cape Town, Cape Town, South Africa; Department of Psychiatry and Mental Health, University of Cape Town, Cape Town, South Africa; Department of Neurology and Department of Psychiatry and Biobehavioral Sciences, University of California, Los Angeles, Los Angeles, California, USA; Department of Bioengineering, University of California, Los Angeles, Los Angeles, California, USA; Department of Neurology and Department of Psychiatry and Biobehavioral Sciences, University of California, Los Angeles, Los Angeles, California, USA; Department of Psychiatry and Mental Health, University of Cape Town, Cape Town, South Africa; The Neuroscience Institute, University of Cape Town, Cape Town, South Africa; Department of Psychiatry and Mental Health, University of Cape Town, Cape Town, South Africa; Medical Research Council Clinical Trials Unit, University College London, London, United Kingdom; Department of Paediatrics and Child Health, Red Cross War Memorial Children's Hospital, University of Cape Town, Cape Town, South Africa; South African Medical Research Council Unit on Child and Adolescent Health, University of Cape Town, Cape Town, South Africa; The Neuroscience Institute, University of Cape Town, Cape Town, South Africa; Department of Psychiatry and Mental Health, University of Cape Town, Cape Town, South Africa; South African Medical Research Council Unit on Risk and Resilience in Mental Disorders, University of Cape Town, Cape Town, South Africa; Department of Paediatrics and Child Health, Red Cross War Memorial Children's Hospital, University of Cape Town, Cape Town, South Africa; The Neuroscience Institute, University of Cape Town, Cape Town, South Africa

**Keywords:** basal ganglia, child brain structure, HIV exposure, immune function, language

## Abstract

**Background:**

Children who are HIV-exposed and uninfected (HEU) are at risk for early neurodevelopmental impairment. Smaller basal ganglia nuclei have been reported in neonates who are HEU compared to HIV-unexposed (HU); however, neuroimaging studies outside infancy are scarce. We examined subcortical brain structures and associations with neurocognition in children who are HEU.

**Methods:**

This neuroimaging study was nested within the Drakenstein Child Health Study birth cohort in South Africa. We compared (T1-weighted) magnetic resonance imaging–derived subcortical brain volumes between children who were HEU (n = 70) and HU (n = 92) at age 2–3 years using linear regression. Brain volumes were correlated with neurodevelopmental outcomes measured with the Bayley Scales of Infant and Toddler Development III.

**Results:**

Compared to HU children, on average children who were HEU had 3% lower subcortical grey matter volumes. Analyses of individual structures found smaller volume of the putamen nucleus in the basal ganglia (−5% difference, *P* = .016) and the hippocampus (−3% difference, *P* = .044), which held on adjustment for potential confounders (*P* < .05). Maternal viremia and lower CD4 count in pregnancy were associated with smaller child putamen volumes. Children who were HEU had lower language scores than HU; putamen and hippocampus volumes were positively correlated with language outcomes.

**Conclusions:**

Overall, children who are HEU had a pattern of smaller subcortical volumes in the basal ganglia and hippocampal regions compared to HU children, which correlated with language function. Findings suggest that optimizing maternal perinatal HIV care is important for child brain development. Further studies are needed to investigate underlying mechanisms and long-term outcomes.

## Teaser

Children who are HIV-exposed and uninfected had smaller subcortical brain volumes, notably in the basal ganglia and hippocampal regions, compared to HIV-unexposed children. Volumes were associated with maternal antenatal viral load and CD4 and correlated with early child language development.

The population of children born to mothers living with human immunodeficiency virus (HIV) who remain HIV-free is expanding, estimated at 16 million worldwide [1]. In some sub-Saharan African countries, >1 in 5 children are born HIV-exposed and uninfected (HEU) [1]. While infant HIV infection is known to affect child development, perinatal exposure to HIV without infection is also a risk factor for adverse outcomes. Children who are HEU have been found to have subtle impairment in early neurodevelopment when compared to their HIV-unexposed (HU) peers [2, 3]. In particular, poorer language outcomes have been noted by 24 months in large studies [4, 5], and results from a recent meta-analysis found that children who are HEU have lower expressive language and gross motor scores compared to HU children across the early years [3].

Understanding the underlying neurobiology may inform prevention and intervention strategies. However, studies examining brain structure of children who are HEU are scarce, and systematic reviews highlight the lack of neuroimaging research [3, 6]. Recently, infants who are HEU were reported to have smaller basal ganglia nuclei compared to HU infants at 2–6 weeks of age in the Drakenstein Child Health Study (DCHS) [7] and another birth cohort study in South Africa [8]; reduced brain volumes were associated with maternal immunocompromise, as measured by lower CD4 cell counts in pregnancy [7], HIV disease severity, and antiretroviral therapy (ART) timing [8]. The basal ganglia nuclei, notably the caudate and putamen, are integral to networks serving cognition and behavior [9, 10], and this subcortical region is known to be vulnerable in people with HIV infection [11]. Separately, a few studies of older children who are HEU have found altered white matter using diffusion tensor imaging as well as differences in neurometabolites on magnetic resonance spectroscopy [12, 13], suggesting that brain development may continue to be affected. However, to our knowledge, there are no studies quantitatively reporting on perinatal HIV exposure and subcortical brain volumes beyond infancy.

The first 3 years of life, from conception through early childhood, are fundamental for brain development, when >80% of subcortical brain growth happens [14]. The age-related changes in brain structure that occur during this period are complex, establishing the foundations of cognitive and language development [15]. In the context of HIV, brain development may be affected by a multitude of factors including HIV and ART exposure, biological factors, an altered uterine environment, and socioenvironmental variables. Understanding the association between early brain structure and function in the context of HIV exposure may help to elucidate underlying mechanisms behind neurodevelopmental impairment. In this study of the DCHS population-based birth cohort, we aimed to investigate whether associations of HIV exposure with subcortical brain structures are evident at age 2–3 years, and whether volumetric differences are related to maternal HIV disease severity and neurodevelopmental outcomes. We hypothesized that children who are HEU would show smaller brain volumes in the basal ganglia region, as reported in infants who are HEU [7], and that these would correlate with neurocognitive function.

## MATERIALS AND METHODS

### Study Design and Participants

The DCHS is a South African birth cohort study established to investigate the determinants of child health [16, 17]. The population has an antenatal HIV prevalence of 21%. Between 2012 and 2015, pregnant women aged 18 years or older attending routine antenatal appointments were recruited from 2 public sector primary healthcare clinics with written informed consent. Mother–child pairs are followed up prospectively.

This nested longitudinal neuroimaging substudy enrolled children from the DCHS [18, 19]. Between 2012 and 2015, a sample of infants aged 2–6 weeks had magnetic resonance imaging (MRI) scans. From 2016 to 2018, these children were invited to return for a further MRI. Additional children aged 2–3 years were invited based on risk factor exposure. The subgroup had specific exclusion criteria of conditions known to impact brain development including prematurity, birth factors (Apgar score <7 at 5 minutes or neonatal intensive care admission), medical comorbidities, maternal use of illicit drugs during pregnancy, child HIV infection, or MRI contraindications. Additional written informed consent was obtained for the neuroimaging.

### Procedures

#### HIV Care

HIV testing of all mothers was performed routinely during pregnancy and the postnatal period, and children were tested at 6 weeks, 9 months, and 18 months and after cessation of breastfeeding [20]. Mothers living with HIV were initiated on ART per local guidelines at the time. Prior to May 2013, mothers were given triple ART or zidovudine monotherapy from 14 weeks’ gestation dependent on WHO clinical stage and CD4 cell count. From 2013, all pregnant women were initiated on triple ART for life. Children with HIV exposure received prophylaxis using nevirapine alone or in combination with zidovudine. We extracted data on maternal CD4 cell count and viral load during pregnancy from folders and the online National Health Laboratory Service system.

#### Sociodemographic Data

Sociodemographic data, birth anthropometry, gestational age, and infant feeding data were collected using standard protocols [21]. Weight, height, and head circumference were measured during the scan visit. Maternal alcohol use during pregnancy was recorded as a composite measure derived from the Alcohol, Smoking and Substance Involvement Screening Test in pregnancy and 2 retrospective self-report questionnaires [17, 18].

### Neuroimaging

#### MRI Data Acquisition and Processing

Structural T1-weighted images were acquired on a 3-Tesla Siemens Skyra scanner at the Cape Universities Body Imaging Centre. Given the need to limit motion for high-quality scans, the imaging was carried out during natural sleep without sedation or general anesthetic. Scans were arranged for the lunchtime nap, or later in the day when the children were more likely to sleep [19]. A 3D MEMPRAGE (multi-echo magnetization prepared rapid acquisition gradient echo) scan in sagittal orientation was used: repetition time 2530 ms, echo time 1.69, 3.54, 5.39, 7.24 ms, inversion time 1100 ms, flip angle 7.0°, voxel size 1.0 mm^3^, 176 slices, field of view: 224 × 224 × 176 mm.

Imaging data processing involved a series of automated steps using FreeSurfer software version 6.0 (http://surfer.nmr.mgh.harvard.edu/) [22] at the Centre for High Performance Computing (Cape Town). Subcortical volume and intracranial volume (ICV) were extracted from segmented images. Parcellation into the 7 subcortical regions (caudate, putamen, pallidum, thalamus, hippocampus, amygdala, nucleus accumbens) was performed [23].

#### Quality Control

Quality control was performed blinded to maternal HIV status. MRI scans were reported by a radiologist; incidental findings were reviewed by a pediatric neurology consultant and appropriate care was arranged using local clinical pathways. Images were visually inspected for artefacts at the outset and segmentation errors were assessed following processing. The ENIGMA protocol for subcortical brain regions (http://enigma.ini.usc.edu/protocols/imaging-protocols/) was used to assess for outliers.

### Neurodevelopmental Assessments

Neurodevelopment was measured using the Bayley Scales of Infant and Toddler Development, third edition (BSID-III) tool, which has been validated in South Africa [24, 25]. Trained assessors blinded to HIV exposure status assessed cognitive, language, and motor outcomes of children by direct observation. Quality control and monitoring were implemented. Age-adjusted composite scores for cognitive, language, and motor domains were used. While neurodevelopment was assessed across the whole DCHS cohort, we report the results for the imaging subsample here.

### Statistical Analysis

We conducted targeted analyses to address our hypotheses. Robust simple and multiple linear regression models were constructed with HIV exposure status as the independent factor and (*i*) total subcortical volume (summing all 7 regions together) and (*ii*) each individual region volume as the dependent factors. Three variables were identified a priori as covariates (age at scan, child sex, and ICV), given their known association with brain structure and to account for overall head (brain) size differences. Given the known influence of other socioenvironmental factors on neurodevelopment, additional covariates (maternal education, maternal age at birth, and household income) were identified using a directed acyclic graph constructed previously [5]. To restrict the number of statistical comparisons when analyzing individual subcortical regions, we used the mean of left and right hemispheres and examined each hemisphere separately where significant associations were identified. Significance level was set to *P* < .05. We calculated Cohen d effect sizes and the percentage difference using the mean absolute difference in volume between children who were HEU and HU relative to the mean HU volume.

To further improve our understanding of the relationship between HIV exposure and neurodevelopment, we next investigated the relationship between subcortical brain volumes and cognitive, language, and motor outcomes using Pearson correlations. We report correlation coefficients and 95% confidence intervals (CIs). Additionally, we explored the associations of maternal CD4 cell count and viral load in pregnancy with subcortical regional volumes that had significant differences by HIV exposure status. Sensitivity analyses were performed (*i*) to investigate how breastfeeding might have influenced the main effect of HIV exposure status on volumetric measures and (*ii*) to check the results held on excluding imaging outliers. All statistical analyses were performed using Stata software version 14.2 (StataCorp, College Station, Texas).

### Data Availability

The de-identified data that support the findings of this study are available from the authors upon reasonable request as per DCHS cohort guidelines.

## RESULTS

### Demographics

**Figure 1.**
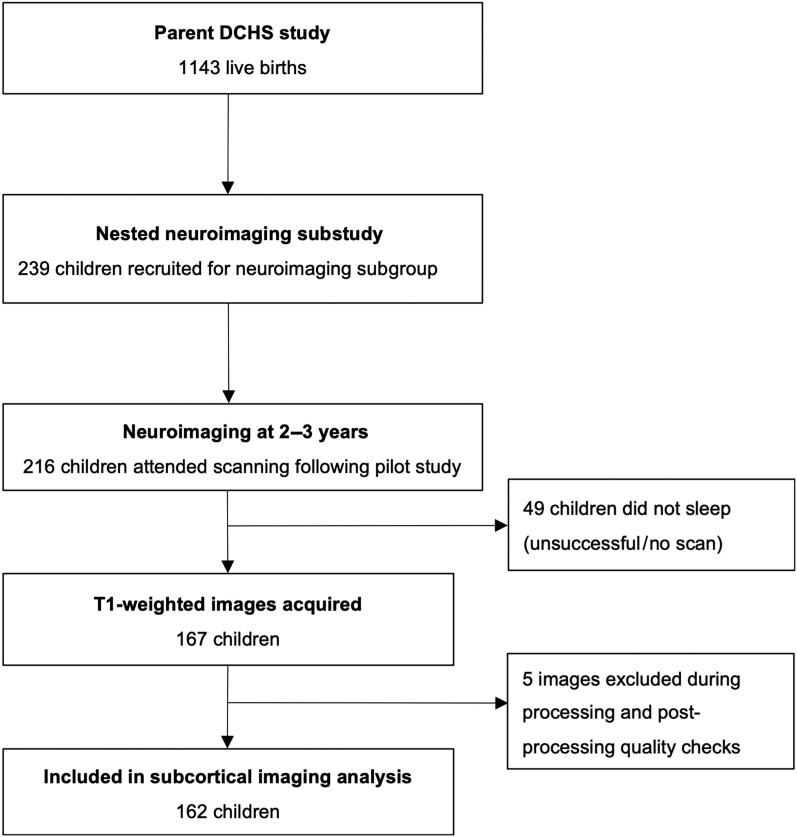
Drakenstein Child Health Study (DCHS) cohort flowchart of children included in this analysis.

A total of 162 children with high-resolution scans were included, 70 HEU and 92 HU (Figure 1). The mean age at scan was 34.1 months (standard deviation, 1.7 months) and 94 (58%) were male. Sociodemographic, clinical, and neurodevelopmental data are reported in Table 1. There were no group differences in maternal socioeconomic parameters and birth outcomes, although mothers with HIV were older (29.6 vs 26.8 years). Mothers with HIV were less likely to breastfeed (40% vs 90%), although mean duration of exclusive breastfeeding was similar (1.6 vs 2.0 months) in those who did commence breastfeeding. Overall, 69 of 70 (99%) children HEU were born to mothers taking triple ART, with the most common regimen being tenofovir, emtricitabine, and efavirenz. More than half of mothers initiated ART in pregnancy, with 44% initiating ART prior to pregnancy. During pregnancy, median maternal CD4 cell count was 476 cells/μL; 23% of mothers had detectable viral loads. Most infants with HIV exposure had nevirapine prophylaxis (80%), while the remaining had nevirapine and zidovudine (Table 2) according to local standard of care.

**Table 1.** Sociodemographic and Clinical Characteristics of Children According to HIV Exposure

Variable	HEU Children (n = 70)	HU Children (n = 92)	*P* Value
Maternal sociodemographic characteristics			
Household income per month (>1000 rand [>∼75 USD])	46 (65.71%)	65 (70.65%)	.50
Maternal education (completed secondary)	19 (27.14%)	35 (38.04%)	.15
Employment status (employed)	17 (24.29%)	27 (29.35%)	.47
Maternal alcohol use during pregnancy	7 (13.46%)	17 (21.25%)	.26
Maternal age at birth, y, mean (SD)	29.57 (4.94)	26.80 (5.81)	.002*
Maternal clinical characteristics			
Maternal hospitalization during pregnancy	3 (4.29%)	2 (2.17%)	.65
Maternal history of TB	7 (10.14%)	4 (4.35%)	.21
Child sociodemographic characteristics			
Child age at scan, mo, mean (SD)	33.83 (1.79)	34.28 (1.65)	.10
Child sex (male)	46 (65.71%)	48 (52.17%)	.08
Gestational age at birth, wk, mean (SD)	38.43 (2.46)	39.17 (2.51)	.06
Any breastfeeding	28 (40.00%)	83 (90.22%)	<.001*
Exclusive breastfeeding	25 (35.71%)	55 (59.78%)	.002*
Exclusive breastfeeding duration, wk, mean (SD)	1.56 (2.09)	2.03 (1.51)	.10
Child neurodevelopmental outcomes			
Language development, mean (SD)	81.82 (10.68)	86.25 (11.84)	.03*
Cognitive development, mean (SD)	85.00 (9.30)	87.44 (9.29)	.12
Motor development, mean (SD)	93.22 (11.38)	94.47 (10.55)	.51
Anthropometry			
Birth head circumference, cm, mean (SD)	33.31 (1.92)	33.90 (1.97)	.06
Birthweight, kg, mean (SD)	3.04 (0.60)	3.14 (0.55)	.29
Low birth weight	10 (14.29%)	9 (9.78%)	.38
Weight at scan, kg, mean (SD)	13.94 (2.02)	13.79 (1.90)	.63
Height at scan, cm, mean (SD)	90.63 (4.04)	91.80 (3.91)	.08
Head circumference at scan, cm, mean (SD)	49.78 (1.76)	49.75 (1.79)	.97
Neuroanatomical variables			
Total intracranial volume, cm^3^, mean (SD)	1208.46 (116.49)	1218.68 (119.14)	.59

Data are presented as No. (%) unless otherwise indicated. Continuous variables were compared with unpaired *t* tests; categorical variables were compared with χ^2^ or Fisher exact test if n < 5. Percentages are cited among those with nonmissing values. Missing data: relationship status (n = 1), alcohol (n = 30), maternal history of TB (n = 1), head circumference at birth (n = 2), head circumference at scan (n = 1), height at scan (n = 21), cognitive composite score (n = 16), language composite score (n = 24), motor composite score (n = 21).

Abbreviations: HEU, HIV-exposed and uninfected; HU, HIV-unexposed; HIV, human immunodeficiency virus; SD, standard deviation; TB, tuberculosis; USD, United States dollars; VL, viral load.

^*^ *P* < .05.

**Table 2.** Maternal and Child HIV Variables

Variable	HEU Children (n = 70)
Maternal HIV diagnosis	
Prior to pregnancy	51 (72.86%)
During pregnancy	19 (27.14%)
Maternal CD4 cell count during pregnancy^a^	
Median (IQR) cell count, cells/μL	476 (344–677)
<350 cells/μL	17 (27.87%)
350–500 cells/μL	16 (26.23%)
≥500 cells/μL	28 (45.90%)
Maternal viral load in pregnancy^a^	
<40 copies/mL	44 (77.19%)
≥40 copies/mL	13 (22.81%)
Maternal ART	
Monotherapy with zidovudine	1 (1.43%)
Triple therapy	69 (98.57%)
Maternal ART initiation	
Prior to pregnancy	31 (44.29%)
During pregnancy	39 (55.71%)
Infant prophylaxis	
Nevirapine alone	56 (80.00%)
Nevirapine + zidovudine	14 (20.00%)

Data are presented as No. (%) unless otherwise indicated. Percentages are cited among those with nonmissing values. Missing data: CD4 count (n = 9), viral load (n = 13).

Abbreviations: ART, antiretroviral therapy; HEU, HIV-exposed and uninfected; HIV, human immunodeficiency virus; IQR, interquartile range.

^a^Where multiple results were available, the lowest maternal CD4 count within 1 year prior to birth and 3 months postbirth was used to reflect maternal immunosuppression in pregnancy and maximize sample numbers. Maternal viral load during pregnancy was classified into <40 (undetectable) and ≥40 (detectable) copies/mL; where there was >1 measurement, we selected the highest as a reflection of the most severe disease exposure.

On examination of neurodevelopmental outcomes, children who were HEU had poorer language outcomes, scoring approximately 5 points lower than HU children (81.8 vs 86.3, *P* = .03). Cognitive (85.0 vs 87.4, *P* = .12) and motor scores (93.2 vs 94.5, *P* = .51) were similar. ICV did not significantly differ across groups (Table 1).

### Subcortical Volumes and HIV Exposure Status

**Figure 2.**
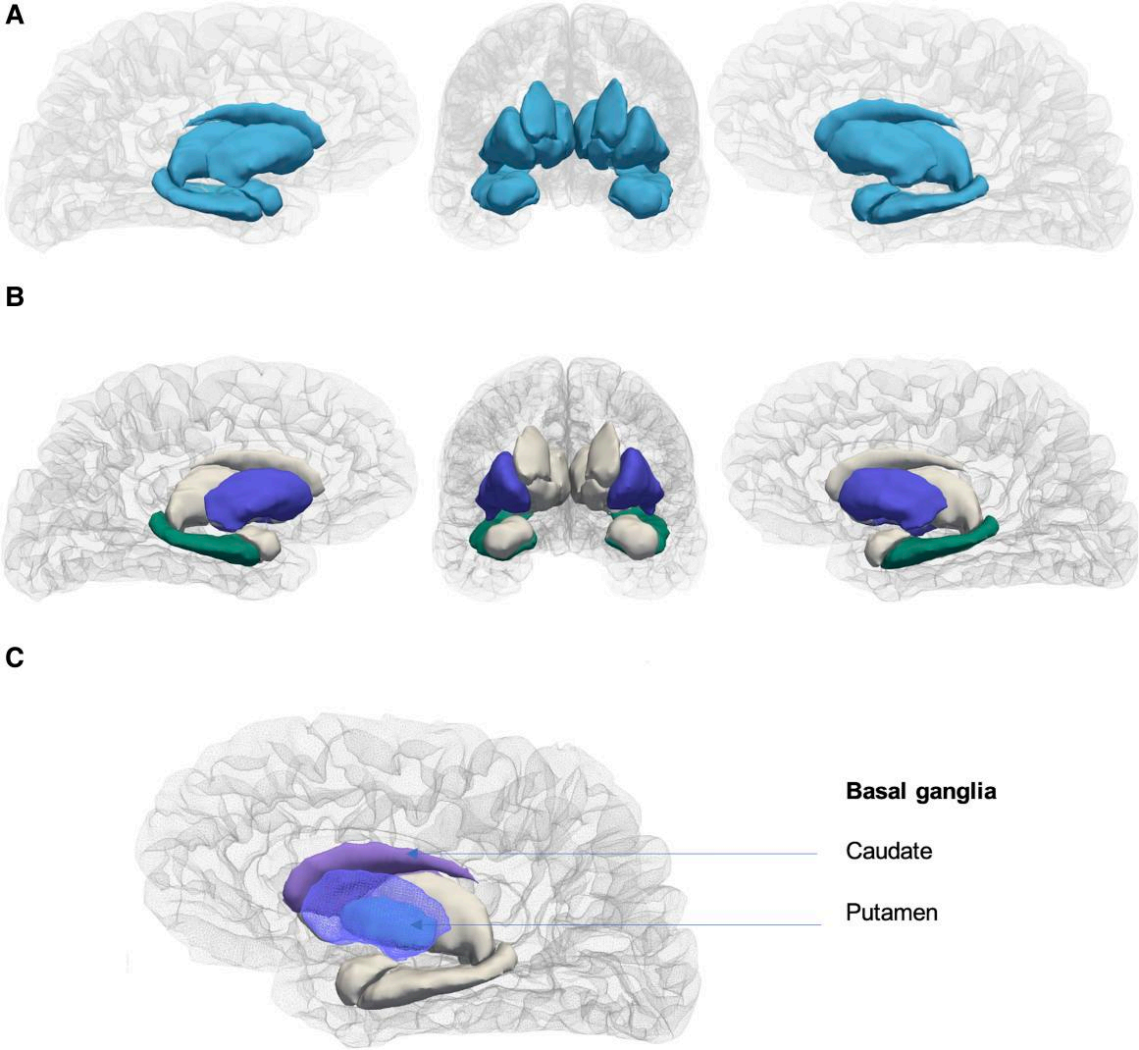
Representation of subcortical structures highlighting those affected in children with perinatal HIV exposure. *A*, All subcortical structures. *B*, Individual structures with significant differences between children who are HIV-exposed and uninfected and HIV-unexposed at 2–3 y. Dark blue: putamen; dark green: hippocampus. *C*, Detailed view of the basal ganglia.

The comparisons of subcortical volumes between children who were HEU versus HU are shown in Table 3 and illustrated in Figure 2. On average, we found that children who are HEU had significantly smaller total subcortical grey matter volume compared to HU children (46 929 vs 48 311 mm^3^, *P* = .049; Cohen d effect size, −0.31 [95% CI, −.63 to −.00]), which represented a difference of 2.9%. The findings held after adjusting for multiple covariates including child age, sex, ICV, maternal education, household income, and maternal age (adjusted mean difference, −945.92 mm^3^ [95% CI, −1774.06 to −117.78 mm^3^], *P* = .025).

**Table 3.** Mean Differences in Subcortical Brain Volumes According to HIV Exposure

Region	Mean (SD) Volume, mm^3^		Unadjusted Difference (95% CI)	*P* Value	Effect Size Cohen d (95% CI)	Fully Adjusted^a^ Difference (95% CI)	*P* Value	Effect Size Cohen d (95% CI)
	HEU (n = 70)	HU (n = 92)						
Total subcortical volume	46 929 (4597)	48 311 (4092)	−1381.89 (−2755.27 to −8.52)	.049*	−0.31 (−63 to −.00)	−945.92 (−1774.06 to −117.78)	.025*	−0.22 (−.53 to .10)
Thalamus	5788 (602)	5919 (548)	−130.51 (−311.86 to 50.85)	.157	−0.23 (−.54 to .09)	−90.87 (−209.98 to 28.24)	.134	−0.16 (−.47 to .15)
Caudate	3246 (525)	3361 (472)	−114.66 (−272.16 to 42.83)	.152	−0.23 (−.54 to .08)	−44.60 (−172.51 to 83.32)	.492	−0.09 (−.40 to .22)
Putamen	4381 (576)	4597 (543)	−215.98 (−391.98 to −39.97)	.016*	−0.37 (−.69 to −.06)	−181.19 (−342.46 to −19.92)	.028*	−0.32 (−.63 to −.00)
Pallidum	1535 (225)	1574 (200)	−39.26 (−106.35 to 27.84)	.250	−0.19 (−.50 to .13)	−19.16 (−77.36 to 39.03)	.516	−0.09 (−.40 to .22)
Hippocampus	3043 (321)	3149 (341)	−106.10 (−209.42 to −2.78)	.044*	−0.31 (−.62 to .00)	−86.34 (−171.80 to −.87)	.048*	−0.26 (−.57 to .06)
Amygdala	1242 (159)	1281 (160)	−38.80 (−88.70 to 11.10)	.127	−0.24 (−.55 to .07)	−31.20 (−77.13 to 14.73)	.182	−0.19 (−.51 to .12)
Nucleus accumbens	587 (90)	572 (83)	14.92 (−12.47 to 42.30)	.284	0.17 (−.14 to .48)	17.15 (−8.63 to 42.93)	.191	0.20 (−.11 to .51)

Regional volumes (mean of left and right hemispheres), mean differences (regression coefficients unadjusted and fully adjusted in multiple regression models), *P* values, and effect sizes for associations between brain volumes and HIV exposure. Residuals were assessed for each model using quantile-quantile plots and were normally distributed.

Abbreviations: CI, confidence interval; HEU, HIV-exposed and uninfected; HU, HIV-unexposed; HIV, human immunodeficiency virus; SD, standard deviation.

^a^Fully adjusted models included child age at scan, child sex, intracranial volume, maternal education, household income, and maternal age.

^*^ *P* < .05.

On examination of individual structures, children who were HEU had lower putamen volume compared to HU children (−4.7%; 4381 vs 4597 mm^3^, *P* = .016; effect size, −0.37 [−.69 to −.06]). Findings held on adjusting for potential confounders (adjusted mean difference, −181.19 mm^3^ [95% CI −342.46 to −19.92 mm^3^], *P* = .028) and results were similar when analyzed separately by hemisphere. Compared to HU, children who were HEU also had lower hippocampal volume (−3.4%; 3043 vs 3149 mm^3^, *P* = .044; effect size, −0.31 [95% CI, −.62 to .00]), a finding that remained on adjustment (adjusted mean difference, −86.34 mm^3^ [95% CI, −171.80 to −0.87 mm^3^], *P* = .048). On analyzing the separate hemispheres (Supplementary Table 1), similar adjusted differences were seen (left hippocampus: −88.11 mm^3^ [95% CI, −169.80 to −6.42 mm^3^], *P* = .035; right hippocampus: −84.56 mm^3^ [95% CI, −183.30 to 14.18 mm^3^], *P* = .093); however the right hippocampus lost statistical significance. There were no significant differences in other subcortical volumes (thalamus, pallidum, amygdala, caudate, nucleus accumbens; all *P* > .05).

### Subcortical Volumes and Neurodevelopmental Outcomes

Next, we explored the correlation between brain structure and cognitive, language, and motor outcomes. The results from the correlation analyses are presented in Table 4. Putamen and hippocampal volumes were found to be positively correlated with language, with modest effect sizes (putamen: *r* = 0.20 [95% CI, .04 to .36], *P* = .017; hippocampus: *r* = 0.21 [95% CI, .05 to .37], *P* = .012). There were no structural correlations with development in other domains.

**Table 4.** Correlations Between Subcortical Brain Volumes and Developmental Domains

Region	Bayley Scales of Infant and Toddler Development								
	Language Development			Cognitive Development			Motor Development		
	*r*	(95% CI)	*P* Value	*r*	(95% CI)	*P* Value	*r*	(95% CI)	*P* Value
Thalamus	0.13	(−.04 to .29)	.121	−0.00	(−.16 to .16)	.999	0.14	(−.03 to .30)	.100
Caudate	0.15	(−.01 to .31)	.073	−0.03	(−.19 to .13)	.708	0.14	(−.03 to .30)	.101
Putamen	0.20	(.04 to .36)	.017*	0.16	(−.00 to .32)	.052	0.13	(−.04 to .28)	.140
Pallidum	0.14	(−.03 to .30)	.111	0.01	(−.15 to .17)	.899	0.06	(−.11 to .22)	.474
Hippocampus	0.21	(.05 to .37)	.012*	0.09	(−.07 to .25)	.283	0.16	(−.00 to .32)	.055
Amygdala	0.09	(−.08 to .25)	.301	0.04	(−.13 to .20)	.647	0.07	(−.09 to .24)	.383
Nucleus accumbens	−0.02	(−.18 to .15)	.860	−0.04	(−.20 to .12)	.641	0.02	(−.15 to .18)	.850

Shown are Pearson correlations and 95% CIs. n = 138 for language development; n = 146 for cognitive development; n = 141 for motor development.

Abbreviation: CI, confidence interval.

^*^ *P* < .05.

### Association With Maternal HIV Disease Severity (CD4 Cell Count and Viral Load in Pregnancy)

**Figure 3.**
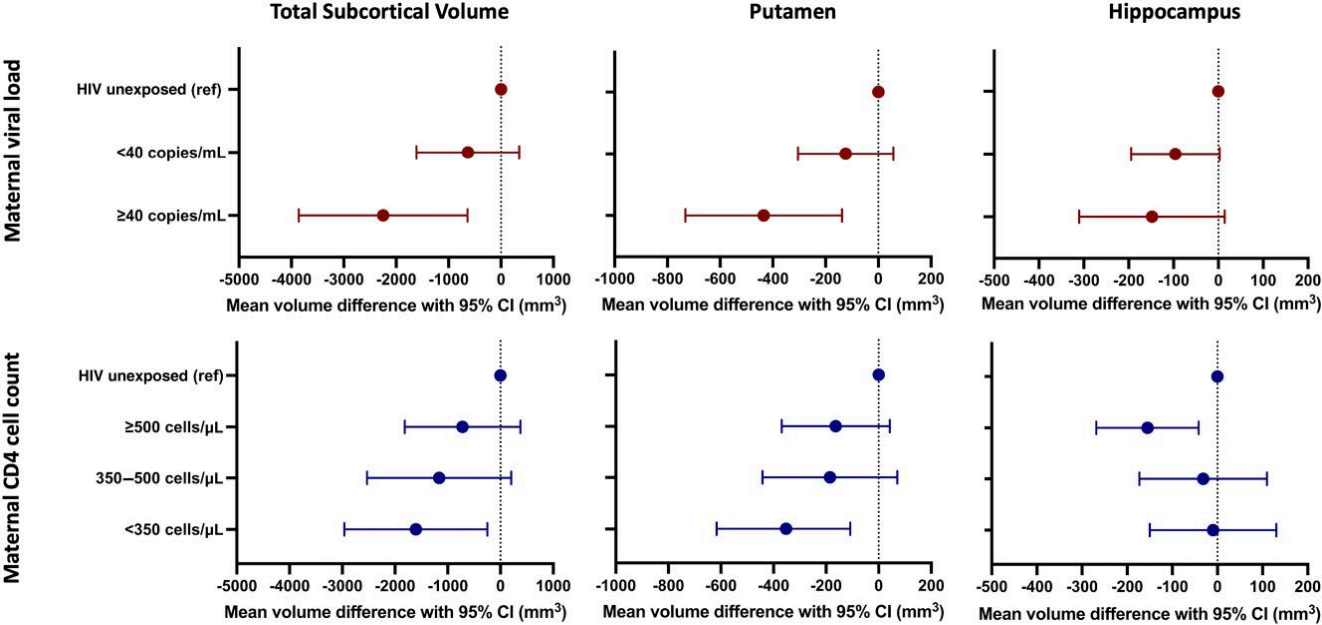
Associations of maternal CD4 cell count and viral load in pregnancy with child total subcortical, putamen, and hippocampus volumes. Mean volume differences are plotted with 95% confidence intervals (CIs) in mm^3^ compared to the HIV–unexposed reference group. Mean volume differences shown are fully adjusted for covariates as detailed in Supplementary Table 2.

In exploratory analyses examining associations between subcortical volumes and maternal CD4 and viral load during pregnancy, we found that lower maternal CD4 (<350 cells/μL) was significantly associated with smaller putamen volumes (Figure 3; Supplementary Table 2). Similarly, maternal viremia (detectable HIV viral load) was associated with smaller total subcortical and putamen volumes with a dose-response pattern across all regions (Figure 3; Supplementary Table 2).

### Sensitivity Analyses

Associations between HIV exposure and smaller putamen and hippocampus volumes remained after adjusting for any breastfeeding and duration of exclusive breastfeeding (Supplementary Table 3*A* and 3*B*). Similar results were also found in sensitivity analyses excluding imaging outliers (Supplementary Table 4).

## DISCUSSION

This study describes the association of perinatal HIV exposure and subcortical brain volumes through early childhood. On average, children who were HEU had smaller subcortical grey matter volumes compared to HU children at 2–3 years, notably in the basal ganglia, building on findings in infancy. Additionally, children who were HEU had lower hippocampal volumes at 2–3 years, a region known to be vulnerable to early-life exposures. These regional subcortical volumes showed small but significant correlations with language outcomes. Overall, the results indicate that HIV exposure in pregnancy may affect early subcortical brain development, with enduring impact and implications for language. Finally, identified associations between maternal CD4 and viral load in pregnancy with subcortical volumes suggest a role for maternal immunosuppression and HIV disease severity in child brain maturation.

The basal ganglia are a collection of nuclei in the deep grey matter, the largest components being the caudate and putamen, which have roles in modulating motor, cognition, behavior, and language processing [9, 10]. At 2–3 years, we found reduced putamen volume in children who are HEU compared to HU. This expands on our prior findings showing smaller caudate volume, the adjoining nucleus, in neonates who are HEU compared to HU [7], as well as the results of another South African study reporting smaller putamen volume in infants who are HEU compared to HU and reduced caudate volume in HEU newborns born to mothers initiating ART in pregnancy [8]. Together the putamen and caudate represent the dorsal striatum of the basal ganglia [26], which is closely linked to the cortex through cortico-striatal pathways [27]. Our results suggest that changes in this region seen in infancy may persist and develop with brain maturation. These findings are consistent with reports of lower basal ganglia metabolites in school-aged children who are HEU [28], qualitative basal ganglia abnormalities in 2-year-old HEU children presenting with neurological symptoms [29], and basal ganglia involvement commonly reported in children with HIV [11].

Individual subcortical structures have varying growth trajectories, and our findings may indicate differential regional vulnerability during windows of brain maturation. The putamen displays earlier maturation relative to other regions, showing the greatest volume change from 3 to 12 months with an approximately 2-fold increase over that period [30]. Increased metabolic activity during periods of maximal growth may make structures more vulnerable to early insults, or the variation associated with these rapid changes may mean that differences are detectable earlier than in other regions. At 2–3 years, we also found lower hippocampus volume in children who are HEU compared to HU. The hippocampus is unique in displaying both neurogenesis and neuroplasticity throughout the postnatal period into adult life [31]. As a result, it is more sensitive to brain insults and stressors than other structures, and smaller left hippocampal volumes have also been seen in children living with HIV [32]. These characteristics underlie the role of the hippocampus in learning and memory formation as well as verbal fluency, and therefore any early deviations from typical maturational trajectories may have implications for later neurocognitive function.

In support of this interpretation, we found an association between subcortical putamen and hippocampus volumes with language development in the present investigation. Neurocognitive functioning is increasingly understood to be dependent on distributed neural networks, which include the basal ganglia and hippocampus [33]. Consistent with this, prior studies have found a relationship between putamen volume, language, and verbal learning [9, 34] and associations between basal ganglia nuclei growth with IQ and language scores [10, 35], potentially indicating early growth of these structures as markers of long-term outcomes. A recent meta-analysis also identified basal ganglia anomalies in developmental language disorders [36]. However, caution needs to be taken when interpreting the clinical implications of brain volume differences, and we note the correlations are small. Altered basal ganglia structure is associated with disrupted cortico-striatal circuitry, which is critical to higher-order cognitive functions, language usage, and learning [37]. Thus, the associations may be due not only to pathology directly related to subcortical volumes, but also to any part of the several parallel loops involving these structures, potentially explaining the smaller effect sizes for the individual subcortical structures.

Early brain development involves myelination, synaptogenesis, pruning, and synaptic modification, which are affected by genetic, biological, environmental, and infectious factors [38]. These processes of brain maturation may be particularly sensitive to direct effects of HIV exposure as well as to the associated effects of the virus on immune activation and inflammatory cytokines [39, 40]. We show an association between lower maternal CD4 count during pregnancy and smaller putamen volume in HEU children, similar to the relationship seen in early infancy [7]. Additionally, we found that maternal HIV viremia was associated with smaller subcortical and putamen volumes. This suggests the basal ganglia may be affected by HIV disease, either directly or via corresponding immune changes. HIV-associated structural deficits, including reduced putamen volumes in infants, have also been shown to be associated with maternal HIV viral load in pregnancy in neonates who are HEU [8], as well as to persist after ART initiation in children with HIV infection [32], suggesting a role for immune activation and/or inflammation in both HIV infection and exposure. Previously we reported a neuroinflammatory pattern on magnetic resonance spectroscopy in HEU children at 2–3 years [12], fitting with this hypothesis.

HIV exposure frequently occurs within the context of a range of risk factors, HIV-related and universal, many of which may affect the developing brain [39]. In particular, the role of ART is important to consider. Most mothers were taking ART regimens including efavirenz. Given that efavirenz has been linked to central nervous system adverse effects in people with HIV [41] and to worse HEU child language outcomes [42], neurotoxicity could be a contributing factor. Dolutegravir is now the recommended first-line regimen and new studies are required to assess integrase inhibitors, considering whether the improved efficacy at reducing viral load may translate to immune protection and better outcomes. We did not find that breastfeeding drove the differences in this analysis, which aligns with data from other fully breastfed cohorts [43]. However, we recognize that the neuroimaging findings need to be replicated in contexts with higher breastfeeding rates. Overall, there are likely multifactorial etiologies, and studies are needed to understand mechanisms and tease apart the effects of HIV, ART, and other socioenvironmental risk.

The findings suggest a few strategies for optimizing outcomes. While the focus needs to remain on prevention of HIV acquisition in mothers and children, the maternal CD4 and viral load results suggest that improved perinatal HIV management to reduce severe disease and immunological compromise may positively impact child neural development. This could include opportunities such as preparation for pregnancy, viral load monitoring, parent education, and ART adherence. The results highlight incentives for early booking in pregnancy to ensure prompt diagnosis and ART initiation, preventing late-presenting HIV disease [44], and the potential role of new antiretrovirals in improving outcomes. Finally, the findings represent a comparison of average group differences, and not all children who are HEU will have developmental difficulties. Therefore, implementing developmental assessments to monitor at-risk children to detect any developmental delay early and refer for interventions will help to ensure optimal health outcomes.

Strengths of this study include the comprehensive neuroimaging and neurodevelopmental assessments; the well-characterized sample with demographically appropriate controls; and the window of measurement in the first 3 years, a critical period of brain growth. However, this study has limitations, some of which are inherent to neuroimaging studies. The relatively small sample size, although large for an MRI study, may have reduced precision and power to detect an effect. Findings need to be replicated, particularly the exploratory analyses, and incorporating a range of immune factors in further research will highlight the full role of immune function. Second, there were some demographic differences between groups in maternal age and breastfeeding. We adjusted for covariates and conducted sensitivity analyses of breastfeeding, which suggested minimal confounding. However, we acknowledge the potential for residual bias and impact of other unmeasured factors, including cytomegalovirus infection and anemia in pregnancy, that need further investigation. Third, ART was not formally explored and the underlying mechanisms need investigation. Finally, we performed cross-sectional analyses, as only 33% of children had scans in infancy and 2–3 years; research is ongoing at older ages so that we will be able to map brain structure longitudinally. Overall, the clinical relevance and trajectories of the identified volumetric differences need to be confirmed in future prospective studies.

In conclusion, at age 2–3 years, children who are HEU showed small differences in subcortical volumes compared to HU children, particularly in the basal ganglia region, similar to those seen in infancy. Our findings represent the first prospective cohort study to report subcortical volumes of children who are HEU at an age when 80% of subcortical growth has occurred [14]. The results suggest possible biological pathways by which HIV exposure might contribute to impaired language development and establish the basal ganglia and hippocampus as regions of vulnerability. Given that the largest part of brain development occurs within the first 3 years of life, influencing future academic and health outcomes, further work will be important to understand contributing mechanisms and how to optimize brain development of this vulnerable population.

## Supplementary Material

ofae317_Supplementary_Data

## Supplementary Data

Supplementary materials are available at *Open Forum Infectious Diseases* online. Consisting of data provided by the authors to benefit the reader, the posted materials are not copyedited and are the sole responsibility of the authors, so questions or comments should be addressed to the corresponding author.

## Notes

***Acknowledgments.*** We acknowledge and thank the children and families of the Drakenstein Child Health Study (DCHS) who made this work possible. We gratefully acknowledge the contributions of study staff in the DCHS, and the radiographers at the Cape Universities Brain Imaging Centre at Groote Schuur Hospital. We also thank the Centre for High Performance Computing (Rosebank) (https://www.chpc.ac.za) for access to its resources.

***Ethical approval.*** This study was approved by the Faculty of Health Sciences, Human Research Ethics Committee, University of Cape Town (401/2009; 525/2012; 044/2017), the Western Cape Department of Provincial Health Research Committee, and the London School of Hygiene and Tropical Medicine Observational/Interventions Research Ethics committee (11903). Written informed consent was obtained at DCHS recruitment, and mothers are reconsented annually. Additional written informed consent was obtained from the parent/guardian at the neuroimaging visit.

***Disclaimer.*** The funders had no role in the study design; data collection, analysis, or interpretation; or report writing.

***Financial support*.** The DCHS study is funded by the Bill & Melinda Gates Foundation (grant number OPP 1017641), the National Research Foundation (NRF), and the National Institutes of Health. Additional support for H. J. Z., D. J. S., and K. A. D. was provided by the South African Medical Research Council. C. J. W. and aspects of the imaging were supported by the Wellcome Trust (grant number 203525/Z/16/Z). K. A. D. and aspects of the research are additionally supported by the NRF; an Academy of Medical Sciences Newton Advanced Fellowship (NAF002/1001) funded by the UK Government’s Newton Fund; the National Institute on Alcohol Abuse and Alcoholism (grant number R21AA023887); the Collaborative Initiative on Fetal Alcohol Spectrum Disorders (developmental grant number U24 AA014811); and the US Brain and Behaviour Research Foundation (Independent Investigator grant number 24467). N. A. G. was supported in this research by a grant from the Carnegie Corporation of New York. A. M. R. is additionally supported by the UK Medical Research Council (MRC) and the UK Department for International Development (DFID) under the MRC/DFID Concordat agreement, which is also part of the EDCTP2 program supported by the European Union (grant reference MR/R010161/1).
