# Supplementary material for: Subcortical Brain Volumes and Neurocognitive Function in Children With Perinatal HIV Exposure: A Population-Based Cohort Study in South Africa
Source: Open Forum Infect Dis. 2024 Jul 17;11(7):ofae317. doi: 10.1093/ofid/ofae317 (PMC11253430; doi:10.1093/ofid/ofae317)
Supplement: ofae317_Supplementary_Data [file ofae317_supplementary_data.pdf]

Supplementary Information

Subcortical brain volumes and neurocognitive function in children with perinatal HIV exposure: a population-based cohort study in South Africa

Catherine J Wedderburn, Shunmay Yeung, Nynke A Groenewold, Andrea M Rehman, Sivenesi Subramoney, Jean-Paul Fouche, Shantanu H. Joshi, Katherine L Narr, Nadia Hoffman, Annerine Roos, Diana M Gibb, Heather J Zar, Dan J Stein,* Kirsten A Donald*

* DJS and KAD are joint senior authors.

This PDF file includes:

Supplementary Table 1: Post hoc hemispheric analyses of regions with a significant association with HIV exposure

Supplementary Table 2: Associations of maternal CD4 cell count and viral load with subcortical regional volumes

Supplementary Table 3: Adjusted mean differences in subcortical brain volumes according to HIV exposure controlling for (a) any breastfeeding and (b) duration of exclusive breastfeeding

Supplementary Table 4: Adjusted mean differences in subcortical brain volumes according to HIV exposure excluding imaging outliers

Supplementary Table 1: Post hoc hemispheric analyses of regions with a significant association with HIV exposure

**Brain volumes**	**Unadjusted difference (95% CI) (n=162)**	**p-value**	**Effect size Cohen's d (95% CI)**	**Fully Adjusted † difference (95% CI)**	**p-value**	**Effect size Cohen's d (95% CI)**
Putamen						
Left hemisphere	-218.70 (-408.29 to -29.10)	0.024*	-0.35 (-0.67 to -0.04)	-188.17 (-361.64 to -8.71)	0.040*	-0.31 (-0.62 to 0.01)
Right hemisphere	-213.26 (-392.69 to -33.82)	0.020*	-0.37 (-0.68 to -0.05)	-174.20 (-339.82 to -8.59)	0.039*	-0.30 (-0.61 to 0.01)
Hippocampus						
Left hemisphere	-110.50 (-211.83 to -9.17)	0.033*	-0.33 (-0.64 to -0.02)	-88.11 (-169.80 to -6.42)	0.035*	-0.26 (-0.58 to 0.05)
Right hemisphere	-101.70 (-214.65 to 11.25)	0.077	-0.28 (-0.59 to 0.04)	-84.56 (-183.30 to 14.18)	0.093	-0.23 (-0.54 to 0.08)

Legend: Mean differences (regression coefficients unadjusted and fully adjusted in multiple regression models), p-values and effect sizes for associations between hemispheric brain volumes and HIV exposure. Effect sizes were calculated using Cohen's d with associated 95% confidence intervals. † Fully adjusted models included child age at scan, child sex, intracranial volume, maternal education, household income, and maternal age. *p<0.05

Supplementary Table 2: Associations of maternal CD4 cell count and viral load with subcortical regional volumes

Brain volume	Maternal CD4 (cells/microlitres)	Mean volume (SD) (mm^3)	*N*	Fully adjusted difference (95% CI)	*p*-value
**Total subcortical brain volume**	HIV-unexposed	48311 (4092)	92	*Reference*	0.065
	≥500	46958 (4211)	28	-718.82 (-1814.66 to 377.02)	
	350-500	46754 (3449)	16	-1162.79 (-2530.19 to 204.62)	
	<350	46380 (6421)	17	-1604.63 (-2959.44 to -249.83)	
**Putamen**	HIV-unexposed	4597 (543)	92	*Reference*	0.028*
	≥500	4389 (546)	28	-163.26 (-368.82 to 42.29)	
	350-500	4380 (472)	16	-185.22 (-441.71 to 71.27)	
	<350	4207 (706)	17	-362.09 (-616.22 to -107.96)	
**Hippocampus**	HIV-unexposed	3149 (341)	92	*Reference*	0.060
	≥500	2959 (287)	28	-154.97 (-268.26 to -41.68)	
	350-500	3102 (235)	16	-31.58 (-172.92 to 109.78)	
	<350	3122 (439)	17	-9.68 (-149.74 to 130.37)	
**Brain volume**	**Maternal viral load, (copies/mL)**	**Mean volume (SD) (mm^3)**	***N***	**Fully adjusted difference (95% CI)**	***p*-value**
**Total subcortical brain volume**	HIV-unexposed	48311 (4092)	92	*Reference*	0.021*
	<40 (undetectable)	47213 (4762)	44	-632.10 (-1612.91 to 348.71)	
	≥40 (detectable)	46081 (5869)	13	-2248.18 (-3858.37 to -638.00)	
**Putamen**	HIV-unexposed	4597 (543)	92	*Reference*	0.014*
	<40 (undetectable)	4447 (594)	44	-123.87 (-304.69 to 56.94)	
	≥40 (detectable)	4176 (590)	13	-434.68 (-731.52 to -137.84)	
**Hippocampus**	HIV-unexposed	3149 (341)	92	*Reference*	0.067
	<40 (undetectable)	3033 (331)	44	-95.78 (-194.59 to 3.04)	
	≥40 (detectable)	3014 (379)	13	-147.97 (-310.19 to 14.25)	

Legend: Total subcortical and regional volumes (mean of left and right hemispheres), mean differences (regression coefficients fully adjusted in multiple regression models), and p-values for associations between brain volumes and maternal CD4 cell count or maternal viral load in pregnancy. Models were adjusted for child age and sex, intracranial volume, household income, maternal age, and education. Regions that had associations with HIV exposure were selected. P-values from the Wald test. *p<0.05.

Supplementary Table 3a: Adjusted mean differences in subcortical brain volumes according to HIV exposure controlling for any breastfeeding

Subcortical brain volumes	Fully Adjusted † difference (95% CI)	P-value	Effect size Cohen's d (95% CI)
Total subcortical volume	-1218.94 (-2153.02 to -284.85)	0.011*	-0.28 (-0.59 to 0.04)
Thalamus	-96.10 (-251.67 to 59.47)	0.224	-0.17 (-0.48 to 0.14)
Caudate	-117.50 (-248.91 to 13.91)	0.079	-0.23 (-0.55 to 0.08)
Putamen	-192.69 (-375.91 to -9.47)	0.039*	-0.34 (-0.65 to -0.02)
Pallidum	-38.81 (-103.79 to 26.16)	0.240	-0.18 (-0.49 to 0.13)
Hippocampus	-122.86 (-225.24 to -20.48)	0.019*	-0.36 (-0.67 to -0.05)
Amygdala	-36.61 (-88.34 to 15.11)	0.164	-0.23 (-0.54 to 0.08)
Nucleus accumbens	10.11 (-19.70 to 39.92)	0.504	0.12 (-0.19 to 0.43)

Legend: Adjusted mean differences, 95% confidence intervals and p-values for associations between brain volumes and HIV exposure accounting for breastfeeding. Fully adjusted models included child age at scan, child sex, intracranial volume, maternal education, household income, and maternal age. *p<0.05. Similar results were seen adjusting for exclusive breastfeeding.

Supplementary Table 3b: Adjusted mean differences in subcortical brain volumes according to HIV exposure controlling for duration of exclusive breastfeeding

Subcortical brain volumes	Fully Adjusted † difference (95% CI)	P-value	Effect size Cohen's d (95% CI)
Total subcortical volume	-1032.04 (-1838.91 to -225.16)	0.013*	-0.24 (-0.55 to 0.08)
Thalamus	-91.61 (-212.61 to 29.38)	0.137	-0.16 (-0.47 to 0.15)
Caudate	-64.55 (191.74 to 62.64)	0.318	-0.13 (-0.44 to 0.18)
Putamen	-183.92 (-342.35 to -25.50)	0.023*	-0.32 (-0.63 to -0.01)
Pallidum	-25.70 (-82.07 to 30.68)	0.369	-0.12 (-0.43 to 0.19)
Hippocampus	-95.30 (-178.76 to -11.83)	0.026*	-0.28 (-0.59 to 0.03)
Amygdala	-32.99 (-78.94 to 12.95)	0.158	-0.20 (-0.52 to 0.11)
Nucleus accumbens	14.60 (-10.88 to 40.07)	0.259	0.17 (-0.14 to 0.48)

Legend: Adjusted mean differences, 95% confidence intervals and p-values for associations between brain volumes and HIV exposure accounting for duration of exclusive breastfeeding. Fully adjusted models included child age at scan, child sex, intracranial volume, maternal education, household income, maternal age and duration of exclusive breastfeeding. *p<0.05. Similar results were seen adjusting for any breastfeeding duration.

Supplementary Table 4: Adjusted mean differences in subcortical brain volumes according to HIV exposure excluding imaging outliers

Brain volumes	Fully Adjusted coefficient (95% CI)	P-value	Effect size Cohen's d (95% CI)
Total volume			
Subcortical grey matter	-876.32 (-1701.85 to -50.78)	0.038*	-0.25 (-0.58 to 0.08)
Subcortical regions			
Thalamus	-66.20 (-191.19 to 58.78)	0.297	-0.13 (-0.46 to 0.20)
Caudate	-55.97 (-194.80 to 82.87)	0.427	-0.12 (-0.45 to 0.21)
Putamen	-169.93 (-320.37 to -19.49)	0.027*	-0.35 (-0.69 to -0.02)
Pallidum	-24.33 (-76.45 to 27.79)	0.358	-0.14 (-0.47 to 0.19)
Hippocampus #	-71.32 (-158.38 to 15.74)	0.108	-0.24 (-0.57 to 0.09)
Amygdala	-30.51 (-73.89 to 12.87)	0.167	-0.22 (-0.55 to 0.11)
Nucleus Accumbens	20.33 (-6.33 to 47.00)	0.134	0.27 (-0.07 to 0.60)

Legend: Adjusted mean differences, 95% confidence intervals and p-values for associations between brain volumes and HIV exposure excluding outliers (n=145). Fully adjusted models included child age at scan, child sex, intracranial volume, maternal education, household income, and maternal age. *p<0.05. #Left hippocampus: -92.04 (-173.80 to -10.28), p=0.028*, effect size -0.32 (-0.65 to 0.01).
